# Quantifying Root Cohesion Spatial Heterogeneity Using Remote Sensing for Improved Landslide Susceptibility Modeling: A Case Study of Caijiachuan Landslides

**DOI:** 10.3390/s25134221

**Published:** 2025-07-06

**Authors:** Zelang Miao, Yaopeng Xiong, Zhiwei Cheng, Bin Wu, Wei Wang, Zuwu Peng

**Affiliations:** 1 School of Geoscience and Info-Physics, Central South University, Changsha 410083, China; zelang.miao@csu.edu.cn (Z.M.); 235007014@csu.edu.cn (Y.X.); 225011035@csu.edu.cn (Z.C.); 2 Laboratory of Geo-Hazards Perception, Cognition and Predication, Central South University, Changsha 410083, China; 3 Bureau of Reclamation of Yunnan Province, Kunming 650216, China; 18879112572@163.com; 4 Geological Survey Institute of Hunan Province, Changsha 410114, China; pengzuwu86@163.com

**Keywords:** spatial heterogeneity, landslide susceptibility, rainfall, root cohesion, slope stability

## Abstract

This study investigates the influence of root cohesion spatial heterogeneity on rainfall-induced landslide distribution across the Loess Plateau, addressing limitations in existing methods that oversimplify root reinforcement. Leveraging Landsat and GaoFen satellite images, we developed a regional root cohesion inversion model that quantifies spatial heterogeneity using tree height (derived from time series Landsat imagery) and above-ground biomass (from 30 m resolution satellite products). This approach, integrated with land use-specific hydrological parameters and an infinite slope stability model, significantly improves landslide susceptibility predictions compared to models ignoring root cohesion or using uniform assignments. High-resolution pre- and post-rainfall GaoFen satellite imagery validated landslide inventories, revealing dynamic susceptibility patterns: farmland exhibited the highest risk, followed by artificial and secondary forests, with susceptibility escalating post-rainfall. This study underscores the critical role of remote sensing-driven root cohesion mapping in landslide risk assessment, offering actionable insights for land use planning and disaster mitigation on the Loess Plateau.

## 1. Introduction

Landslides are among the most frequent geological disasters globally, posing a significant hazard to human life and property [1,2]. In the context of global warming, extreme weather events have become more frequent, with a notable increase in intense rainfall occurrences worldwide [3,4,5]. This trend is particularly evident in the Loess Plateau of China, a region characterized by thick loess deposits and high vulnerability to rainfall-induced landslides. For instance, in Ji County, Shanxi Province, located in the western Loess Plateau, surface loess coverage reaches several tens of meters. Between 3 and 6 October 2021, the region experienced extreme rainfall, triggering over 100 shallow landslides in the Caijiachuan watershed, which severely impacted the livelihoods and economic activities of local residents. While this is a regional case, it reflects a growing global concern, as similar conditions are becoming increasingly common in other parts of the world. Therefore, understanding the susceptibility of superficial landslides under extreme rainfall conditions is essential for both regional disaster prevention and the development of globally applicable risk assessment frameworks [6,7,8,9].

Landslide susceptibility assessment remains a key area of research in disaster prevention and mitigation, serving as the foundation for slope stability analysis and regional planning [10]. This field is typically divided into two main approaches: data-driven methods and physically-based modeling [11,12]. Data-driven models, such as machine learning and deep learning algorithms, rely on extracting features from historical data and training algorithms to predict landslide probabilities. These models are particularly effective at capturing complex nonlinear relationships and can achieve high predictive accuracy when sufficient data are available [13]. Recent studies have demonstrated the versatility of data-driven models in diverse geographic and geomorphological contexts. For example, multi-sensor fusion techniques using UAV photogrammetry and LiDAR have shown promising results in deformation monitoring for landslide-prone terrain [14,15]. Similarly, optical and radar-based remote sensing methods have proven instrumental in disaster management, enabling rapid assessment of landslide impacts [16,17]. Moreover, satellite remote sensing provides an efficient approach for large-scale environmental monitoring, enabling the assessment of spatiotemporal variations in key indicators across diverse terrains. The feasibility of applying satellite-based machine learning for high-resolution environmental analysis at national scales has been demonstrated, supporting its potential in enhancing landslide hazard mapping and early warning systems. Additionally, geostatistical and machine learning approaches have been effectively applied in regions such as Northern Morocco [18] and sparsely vegetated watersheds [19], reinforcing the applicability of data-driven modeling in both hazard mapping and multi-hazard land management. In contrast, physically-based methods emphasize the underlying physical processes governing landslide occurrence. These methods explicitly account for dynamic triggering factors—such as rainfall infiltration and pore water pressure variation over time—as well as static inherent factors related to the terrain, including geological structures, soil properties, and slope geometry. By simulating the interactions among these variables, physically-based models offer a mechanistic understanding of landslide susceptibility [20]. These approaches often use Geographic Information Systems (GISs) to integrate data and develop predictive models. Compared to data-driven techniques, physically-based models offer advantages in terms of mechanistic insight, model interpretability, the ability to handle data scarcity, and the capacity to simulate extreme events [21]. Notable examples of physically-based landslide susceptibility models include TRIGRS [22], SHALSTAB [23], and SINMAP [24]. These models integrate hydrological simulations with infinite-slope stability principles, utilizing ArcGIS to extract terrain features for landslide risk assessment under rainfall conditions. The applicability of susceptibility and hazard zoning methods depends on spatial scale, input data resolution, and planning objectives [25]. While SHALSTAB and SINMAP are effective for the regional assessment of shallow landslides on simple terrain, they rely on steady-state assumptions and have limited parameterization [26,27,28]. In contrast, TRIGRS simulates transient rainfall infiltration and unsaturated soil mechanics, enabling the dynamic assessment of rainfall-induced landslides [29]. Given that the study area is characterized by shallow landslides primarily triggered by typhoon rainfall and key hydrological and geotechnical inputs are available at a 30 m resolution, TRIGRS provides a suitable and physically-based framework for modeling landslide susceptibility here [28].

Root cohesion is a critical parameter in physically-based landslide susceptibility models [30,31,32]. Research has shown that root strength plays a pivotal role in landslide stability, particularly in mountainous regions, where plant roots influence slope stability through various mechanisms [33]. The role of roots in slope stability involves complex biological, mechanical, and hydrological interactions. Trees, shrubs, and herbaceous plants each contribute differently to slope stability due to variations in root structure [32]. Trees generally have deep, extensive root systems, which provide strong anchoring over large areas; shrubs, with shallower but numerous roots, increase soil contact area significantly; and herbaceous plants, with roots concentrated in the topsoil, are crucial for stabilizing the surface layer. In this field, Wu et al. [34] investigated the effect of forest cover on slope stability through laboratory and field tests, measuring soil pore water pressure, shear strength, and other soil properties. They developed a soil–root model to assess tree root contributions to shear strength, finding that forest cover removal significantly reduced shear strength, thereby increasing landslide risk.

In addition to field-based observations, remote sensing and simulation studies further support the complex role of vegetation. Evans et al. [35] using airborne LiDAR data in the Ikawa watershed of Japan, revealed a positive correlation between tree height and root depth, with taller trees contributing more significantly to slope stabilization and reducing landslide frequency. Masi et al. [32] developed a shallow landslide stability model incorporating root effects and Monte Carlo simulations, concluding that root reinforcement is closely linked to rainfall intensity and soil saturation. Gong et al. [36] proposed a regional landslide stability model that integrates rainfall and root effects, calculating root reinforcement using the RipRoot model and incorporating root cohesion into the SINMAP model, which significantly enhanced prediction accuracy. Emadi-Tafti et al. [37] developed the SSHV-2D model, which integrates hydrological and vegetation effects, finding that matric suction in the unsaturated zone and high-density vegetation substantially improved the slope safety factor. Xue et al. [38] improved traditional loess landslide susceptibility models by introducing a quantitative physical model-based evaluation method, confirming that vegetation effectively mitigates shallow landslides and improves model accuracy. However, most root reinforcement models assign fixed values based on vegetation type, overlooking the spatial heterogeneity of root reinforcement within the same land use type, as well as the influence of dynamic factors, such as site conditions and vegetation growth, on root strength. This limitation diminishes the accuracy and reliability of landslide susceptibility assessments.

To investigate the influence of soil–root force spatial heterogeneity on the spatiotemporal distribution of rainfall-induced landslides, this study proposes a new regional root strength inversion model. The model accounts for spatial variations in root forces and estimates root strength using data such as tree height and above-ground biomass. Additionally, it incorporates differences in hydrological parameters across land use types to assess landslide susceptibility during rainfall events using the infinite slope stability model. The model is applied at the catchment scale, with simulations performed on a 30 m-resolution grid covering the entire Caijiachuan Watershed, Shanxi, China. Each grid cell is treated as an independent slope unit, allowing for regional-scale assessment while preserving local slope stability characteristics. The structure of this paper is as follows: Section 2 introduces the study area and data sources. Section 3 presents the methodology, including the root cohesion inversion model and slope stability analysis. Section 4 shows the results and comparative analysis of different scenarios. Section 5 concludes the study with key findings and future perspectives.

## 2. Study Area and Data Sources

### 2.1. Study Area

**Figure 1:**
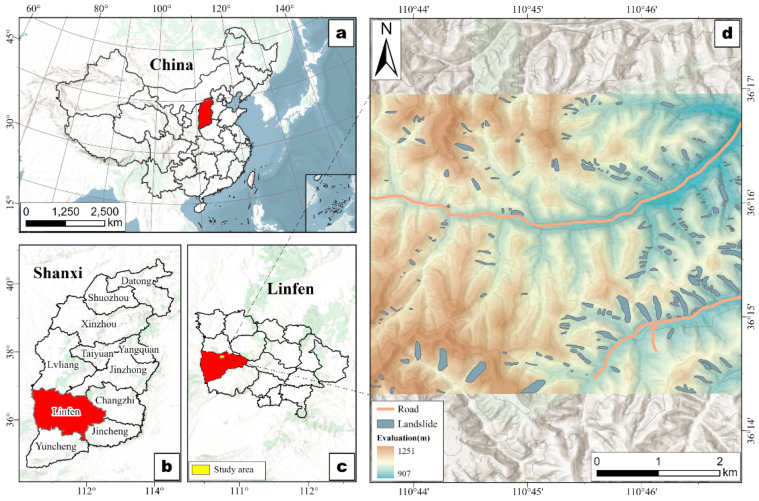
Map of the study area. (**a**) Map of China; (**b**) map of Shanxi Province; (**c**) map of Linfen City; and (**d**) DEM and the landslide inventory of the study area.

The study area is situated in the western part of Linfen City, Shanxi Province, within the southwestern Loess Plateau (35°53′10″ N–36°21′18″ N, 110°26′41″ E–111°07′34″ E), as illustrated in Figure 1. The region is characterized by significant elevation variation, ranging from 907 m to 1251 m above sea level, resulting in pronounced topographic relief, as illustrated in Figure 1d. The terrain exhibits an average slope of 19°, with a maximum slope reaching 52° and a slope variance of 8, indicating high spatial heterogeneity in slope gradients. Its complex and fragmented terrain, typical of the Loess Plateau, features steep slopes, deeply incised valleys, and broad terraces. The dominant soil type is Calcic Cambisols, as per FAO soil classification [39]. The area experiences a temperate continental monsoon climate, with an average annual temperature of approximately 10.5 °C and an average annual precipitation of around 704 mm, of which 64.2% falls between July and October. Annual potential evaporation averages 1723.9 mm, with over half occurring between April and July. Monthly evaporation typically exceeds precipitation, especially during this period, reflecting the arid conditions typical of the Loess Plateau [40]. Meteorological data for temperature, precipitation, and evaporation were obtained from the China Meteorological Administration’s authoritative dataset (https://data.cma.cn, accessed on 8 May 2024). These climatic conditions strongly influence soil erosion and gully development. High evaporation rates, coupled with concentrated rainfall patterns, lead to rapid surface water loss. Intense rainfall events during the rainy season generate substantial surface runoff, exacerbating soil erosion, especially on bare slopes and exposed surfaces.

### 2.2. Data Sources Used in This Study

The data utilized in this study include basic geographic information, rainfall records, soil properties, and vegetation characteristics, as detailed in Table 1. Key parameters for the slope stability assessment model include soil density, internal friction angle, soil cohesion, vegetation root strength, slope, saturated hydraulic conductivity, upslope contributing area, and rainfall. For the loam-dominated soils of the study area, the soil density, internal friction angle, and cohesion are set at 1430 kg/m^3^, 29°, and 14 kPa, respectively. The slope and upslope contributing area are derived from the digital elevation model (DEM), while soil thickness data are sourced from the Oak Ridge National Laboratory Distributed Active Archive Center.

### 2.3. Description and Analysis of Landslide Inventory

**Figure 2:**
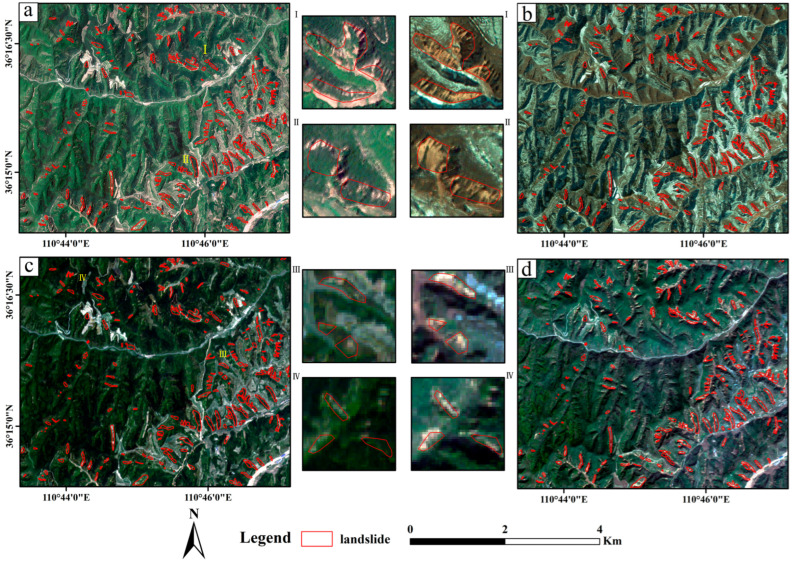
(**a**,**c**) are satellite image from August 2021 (pre-rainfall). (**b**,**d**) are satellite imagery from November 2021 (post-rainfall). Regions I, II, III, and IV denote typical landslide-affected areas.

Intense rainfall impacted the study area from 3–6 October 2021. To investigate the resulting landslides, the high-resolution remote sensing imagery acquired closest to the rainfall event, both before and after, was analyzed. The datasets included GaoFen-2 imagery provided by the China Centre for Resources Satellite Data and Application (https://www.cpeos.org.cn/, accessed on 8 May 2024) and Sentinel-2 Level-2A imagery downloaded from the Copernicus Open Access Hub (https://www.copernicus.eu/, accessed on 8 May 2024). The satellite data were captured in August 2021 (pre-rainfall) and November 2021 (post-rainfall), covering the entire study area with minimal cloud cover (Figure 2a,c: pre-rainfall GaoFen and Sentinel-2; Figure 2b,d: post-rainfall GaoFen and Sentinel-2).

Landslide mapping was performed through manual photo-interpretation following visual characteristics such as surface disturbances, bare soil exposure, and slope morphology changes. Only rainfall-induced shallow translational landslides were delineated in this study, while other types of mass movements (e.g., debris flows) were not included. A total of 240 shallow landslides were identified, covering approximately 1.047 km^2^ within the ~28.2 km^2^ study area. These events were confined to the rainfall period and do not include pre-existing or historical landslides, as the objective was to assess the short-term response of slope stability to a specific extreme rainfall event. Figure 2 highlights a concentration of landslides in the southeastern portion of the study area. The smallest landslide area (21,827 m^2^) was observed within the secondary forest sub-basin, followed by the artificial forest sub-basin (39,135 m^2^). The largest landslide area (51,295 m^2^) occurred in the farmland sub-basin.

**Figure 3:**
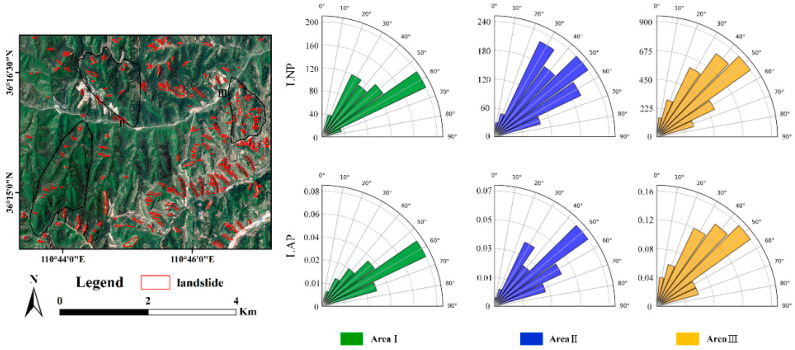
Distribution characteristics of landslide in two catchments under different slope.

The distribution characteristics of landslides across different areas were analyzed using the landslide number density (LNP) and landslide area percentage (LAP). Landslide number density refers to the number of landslides per square kilometer within a given area, providing insight into the frequency of landslide events. Landslide area percentage represents the proportion of the total area affected by landslides, reflecting the spatial extent of landslide impact. Area I represents secondary forest, Area II represents artificial forest, and Area III represents farmland. Landslide distribution varied significantly across slope gradients among the land use types (Figure 3). In farmland and artificial forest areas, landslides were predominantly found on slopes between 35° and 60°, whereas in secondary forest areas, they were mainly distributed on slopes between 40° and 65°. LNP followed a similar trend, with farmland exhibiting the highest densities. LAP values for farmland were markedly higher on slopes ranging from 45° to 60°, while artificial forest areas showed elevated LAP values between 50° and 55°. In secondary forest (or closed forest) areas, the highest LAP values were observed on slopes between 60° and 65°. Additionally, both LNP and LAP in farmland areas were more than double those in artificial forest and secondary forest areas. Statistical analysis revealed that LNP, LAP, and erosion amounts across slope gradients followed the hierarchy of farmland > artificial forest > secondary forest, with steeper slopes experiencing greater impacts.

## 3. Methodology

To examine the influence of land use on the spatiotemporal distribution of rainfall-induced landslides, a novel regional root cohesion model was developed. This model accounts for the spatial variability of root cohesion by incorporating data such as tree height and above-ground biomass to estimate root strength effectively. Additionally, an infinite slope stability model was employed to assess landslide susceptibility during rainfall events, integrating the distinct hydrological parameters associated with various land use types. This approach enabled a detailed analysis of the spatial variations in rainfall-induced landslides across the Loess Plateau under the influence of differing land use practices.

### 3.1. The Infinite Slope Stability Model

The infinite slope stability equation is a theoretical model used to assess slope stability, particularly suitable for analyzing the stability of long, uniform soil slopes. The model assumes that the properties of the slope material are uniformly distributed along the entire slope and that the potential sliding surface beneath the slope is planar. Infinite slope stability analysis primarily focuses on the possibility of soil sliding downward along the slope under the influence of gravity [41,42]. The infinite slope stability equation is derived from the Mohr–Coulomb failure criterion, which predicts the safety factor (FS) of an infinite plane based on the ratio of the stabilizing forces (cohesion and friction reduced by pore water pressure) to the destabilizing forces [29]. The safety factor for the infinite slope model is expressed as follows in Equation (1):

(1) $$FS=\frac{\tan\phi^{\prime }}{\tan\delta }+\frac{C^{\prime }+\psi (Z,t)\gamma_{w}\tan\phi^{\prime }}{\gamma_{s}Z\sin\theta \cos\theta }$$

where $C^{\prime }\left(kPa\right)$ is the effective cohesion of the soil; $\delta \left({}^{\circ}\right)$ is the slope; $\gamma_{s}\left(\mathrm{kg}\cdot m^{-3}\right)$ is the bulk density of the soil; $z\left(m\right)$ is the soil depth; $\phi^{\prime }$(°) is the friction angle of the soil; and ψ is the pressure head determined by the soil depth and rainfall time. It can be seen that the infinite slope stability model calculates FS by considering the ratio of the stabilizing forces induced by soil cohesion and root strength of vegetation to the destabilizing forces caused by the reduction in pore water pressure and the effect of gravity.

### 3.2. The Infiltration Model

The infiltration model employs an analytical solution of a partial differential equation to simulate the infiltration process. For wet initial conditions, the model is based on Iverson’s linearized solution to the Richards equation. This solution incorporates both steady-state and transient components of flow [43]. The steady-state infiltration is determined by the initial water table depth and the steady infiltration rate; under steady infiltration conditions, the slope remains stable and is not at risk of landslides. Transient infiltration, on the other hand, arises from changes in transient pore water pressure caused by short-duration rainfall events. In general, higher rainfall intensity leads to greater transient infiltration [44]. The generalized solution in TRIGRS for pore-water pressure under a finite-depth impermeable boundary condition is provided as Equation (2).

(2) $$\begin{array}{l} \psi (Z,t)=(Z-d)\beta +2\sum_{n=1}^{N}\frac{I_{nz}}{K_{s}}H(t-t_{n}){\left[D_{1}(t-t_{n})\right]}^{\frac{1}{2}}•\\ \sum_{m=1}^{\infty }\left\{ierfc[\frac{\left(2m-1)d_{LZ}-(d_{LZ}-Z\right)}{2{\left[D_{1}(t-t_{n})\right]}^{\frac{1}{2}}}+ierfc[\frac{\left(2m-1)d_{LZ}+(d_{LZ}-Z\right)}{2{\left[D_{1}(t-t_{n})\right]}^{\frac{1}{2}}}]\right\}\\ -2\sum_{n=1}^{N}\frac{I_{nz}}{K_{s}}H(t-t_{n+1}){\left[D_{1}(t-t_{n+1})\right]}^{\frac{1}{2}}•\\ \sum_{m=1}^{\infty }\left\{ierfc[\frac{\left(2m-1)d_{LZ}-(d_{LZ}-Z\right)}{2{\left[D_{1}(t-t_{n+1})\right]}^{\frac{1}{2}}}+ierfc[\frac{\left(2m-1)d_{LZ}+(d_{LZ}-Z\right)}{2{\left[D_{1}(t-t_{n+1})\right]}^{\frac{1}{2}}}]\right\} \end{array}$$

Here, ψ is the groundwater head; t is the rainfall time; z is the depth below the ground surface; d is the steady-state water level depth in the z-direction; $\beta =\cos^{2}\delta (I_{ZLT}/K_{s})$, $K_{S}$ is the saturated hydraulic conductivity, and $I_{ZLT}$ is the initial surface flux. δ is the slope; $d_{LZ}$ is the vertical depth of the impermeable base boundary; $H(t-t_{n})$ is the Heaviside step function, where $t_{n}$ represents the time of the n-th interval in the rainfall infiltration sequence; $D_{0}$ is the hydraulic diffusion coefficient; N is the total number of time intervals; and ierfc(η) is the first-order integral of the Gaussian complementary error function [45], which can be expressed as Equation (3):

(3) $$ierfc(\eta )=1/\sqrt{\pi }\times \exp(-\eta^{2})-\eta \times ierfc(\eta )$$

where the first term represents the steady-state component, while the remaining terms represent the transient components. Previous studies have shown that large-scale landslides are often associated with rainfall events [29]. Among all the variables involved in the model, rainfall is the most rapidly changing factor over short time periods. The model represents the infiltration as the sum of rainfall and the total runoff from the upslope grid cells, as shown in Equation (4), with the infiltration rate not exceeding the saturated hydraulic conductivity:

(4) $$I=P+R_{u}$$

When the infiltration exceeds the saturated hydraulic conductivity, the actual infiltration is calculated using Equation (5):

(5) $$I=K_{s}$$

If $P+R_{u}>K_{s}$, the excess is considered as runoff $R_{d}$, as calculated by Equation (6).

(6) $$R_{d}=P+R_{u}-K_{s}$$

If $P+R_{u}<K_{s}$, then $R_{d}$ is expressed as Equation (7):

(7) $$R_{d}=0$$

### 3.3. The Root Cohesion Inversion Model

Root cohesion ($C_{r}$) plays a critical role in enhancing slope stability and is a critical parameter in the infinite slope stability model. However, existing methods typically estimate root cohesion through laboratory or in situ pull tests, which fail to fully capture the spatial and temporal variability of root cohesion [33]. To address this limitation, the present study examines the relationship between forest stand characteristics, environmental factors, and root force [30]. By establishing regression models based on pull test data, we develop a regional root force inversion model that more accurately reflects the spatiotemporal variability of root cohesion. The derivation process is outlined as follows. Root cohesion in vegetation is conceptualized as the integral of the rooting force at various soil depths. Specifically, it is computed by summing the shear strength provided by the root system at each depth, thereby capturing the overall contribution of vegetation to soil stability. The calculation is expressed in Equation (8):

(8) $$C_{r}=\int_{0}^{\infty }C_{r}(z)\cdot dz$$

where $C_{r}\left(z\right)$ represents the root force at a soil depth of z(m). Root cohesion is a composite measure of the tensile strength of individual roots, influenced by multiple factors such as the tensile strength of single roots, root quantity, distribution, diameter, and material properties. While the tensile strength of individual roots forms the foundation of root force, the overall root force is also determined by the structural arrangement and distribution of the root system. Thus, $C_{r}\left(z\right)$ can be expressed as Equation (9):

(9) $$C_{r}(z)=K\cdot T_{r}\cdot A\left(z\right)$$

where K is a constant, typically assumed to be 1.2 $m^{-1}$ [34]; $T_{r}$ ($N/m^{2}$) represents the tensile strength of an individual root, and $A\left(z\right)$ denotes the root area at a soil depth of z. Typically, the distribution of root area with increasing soil depth follows an exponential decay pattern, which can be represented mathematically as shown in Equation (10):

(10) $$A\left(z\right)=A_{0}e^{-bz}$$

where $A_{0}$ represents the root area in the surface soil and b is the decay coefficient, which reflects the rate at which root area decreases with depth. Therefore, the equation for root cohesion can be rewritten as Equation (11):

(11) $$C_{r}=\int_{0}^{\infty }K\cdot T_{r}\cdot A_{0}e^{-bz}\cdot dz=K\cdot T_{r}\cdot \int_{0}^{\infty }A_{0}e^{-bz}\cdot dz=K\cdot T_{r}\cdot V_{r}=K\cdot T_{r}\cdot \frac{A_{0}}{b}$$

where $V_{r}$ represents the root volume. The tensile strength $T_{r}$ of the root is positively correlated with the tree diameter D, although this relationship is influenced by factors such as species characteristics, root diameter, soil conditions, and growth environment. This relationship is typically expressed using a power function, as shown in Equation (12):

(12) $$T_{r}=\alpha \cdot D^{\beta }$$

where α and β are empirical parameters, with values of 84.2 and 1.31, respectively, as established by previous studies [46]. Direct measurement of tree diameter requires in situ measurements of each tree, which is time-consuming and labor-intensive, especially in large or inaccessible forested areas. For larger study regions, direct measurement of tree diameter becomes impractical, particularly in areas with complex terrain or limited access. Therefore, canopy height (H) can be measured using satellite data, and tree diameter can be inferred using a tree growth model. Thus, tree diameter can be expressed as Equation (13):

(13) $$D=0.00093\times H^{2}+1.18209\times H-0.6642$$

Although it is challenging to directly measure the root area in the surface soil over large areas, this value can be indirectly obtained through the relationship between underground biomass and the root area in the surface soil. Given that the ecosystem in the study area is primarily composed of plant roots, this study equates underground biomass ($B_{r}$) with root mass ($M_{r}$). The root mass is given by Equation (14):

(14) $$B_{r}\approx M_{r}=\rho_{r}V_{r}=\rho_{r}\frac{A_{0}}{b}$$

where root density ($\rho_{r}$) is assumed to be constant within a certain range, with an empirical value of 435.33 $\mathrm{kg}/m^{3}$ used in this study [46]. Previous research has shown that underground biomass ($B_{r}$) is related to aboveground biomass ($A_{r}$) [47] as follows in Equation (15):

(15) $$B_{r}=0.26\times A_{r}$$

By utilizing publicly available aboveground biomass datasets, underground biomass can be inferred through empirical relationships. This provides a basis for determining root cohesion $A_{0}$, as expressed in Equation (16):

(16) $$A_{0}=B_{r}\cdot b/\rho_{r}$$

By combining Equations (11) and (16), we obtain Equation (17):

(17) $$C_{r}=K\cdot T_{r}\cdot \frac{B_{r}}{\rho_{r}}$$

### 3.4. The Infinite Slope Stability Model Considering the Influence of Land Use and Rainfall

Substituting the estimated rainfall infiltration and the derived root cohesion into Equation (1) yields the modified expression presented in Equation (18):

(18) $$FS=\frac{\tan\phi^{\prime }}{\tan\delta }+\frac{C_{r}+C_{s}+\psi (Z,t)\gamma_{w}\tan\phi^{\prime }}{\gamma_{s}Z\sin\theta \cos\theta }$$

where $C_{s}$ is the soil cohesion.

Table 2 presents the saturated hydraulic conductivity for different land uses and Table 3 shows the geotechnical parameters for each soil type. Based on the *FS* values, this study classifies the landslide susceptibility into five categories: very high susceptibility (*FS* ≤ 0.8), high susceptibility (0.8 < *FS* ≤ 1.0), moderate susceptibility (1.0 < *FS* ≤ 1.2), low susceptibility (1.2 < *FS* ≤ 1.5), and very low susceptibility (*FS* > 1.5).

## 4. Experimental Results

### 4.1. Analysis of Root Cohesion Inversion Results

**Figure 4:**
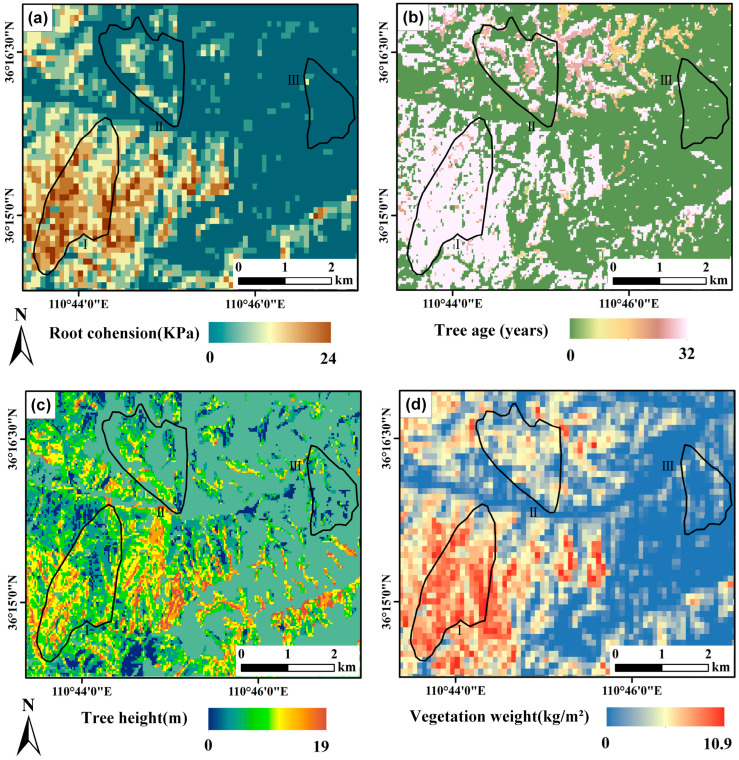
Maps of the study area showing (**a**) root cohesion, (**b**) tree age, (**c**) tree height, and (**d**) above-ground vegetation biomass.

The results of the root cohesion inversion are presented in Figure 4, revealing distinct spatial variations. Root cohesion is notably higher in the southwestern portion of the study area and lower in the northeastern region. These distributions highlight significant contrasts in root cohesion characteristics between vegetation cover types. In the agricultural land sub-watershed, the root cohesion distribution is left-skewed, reflecting relatively weak root strength. This weakness is primarily attributed to the predominance of short-duration seasonal crops, such as wheat and corn, which typically grow for only three to four months and develop shallow root systems. Due to their brief root residence time in the soil, these crops contribute minimally to root cohesion, offering limited mechanical reinforcement to slope stability. These characteristics hinder the development of deep, robust root networks. Furthermore, agricultural practices such as plowing and harrowing disrupt soil structure, reducing compaction and impeding root expansion. These activities also fragment the natural layering and continuity of the soil, diminishing the anchoring capacity of roots. Consequently, the root cohesion of agricultural land vegetation is generally low. In contrast, the planted forest sub-watershed exhibits a more centered root cohesion distribution, indicative of moderate strength. Planted forests are often composed of fast-growing species, such as fir and eucalyptus, selected through scientific management practices. These species typically develop relatively well-structured root systems, with deep-rooted species forming networks that penetrate the soil effectively. However, compared to secondary forests, planted forests tend to have lower species diversity and simpler root architectures, limiting their overall cohesion potential. Additionally, the uniform stand age of planted forests often results in the absence of mature deep roots, which could otherwise enhance root complexity and stability.

The secondary forest sub-watershed, on the other hand, demonstrates a right-skewed root cohesion distribution, signifying strong root strength. Secondary forests are characterized by a high diversity of native tree species, which contribute to a complex and extensive root network. These roots penetrate soil layers more effectively, improving shear resistance and soil stability. Moreover, the older trees typically found in secondary forests possess mature and well-developed root systems, further reinforcing the soil. The diverse and interwoven root structures in these forests provide superior anchoring and stabilization, significantly enhancing root cohesion.

**Figure 5:**
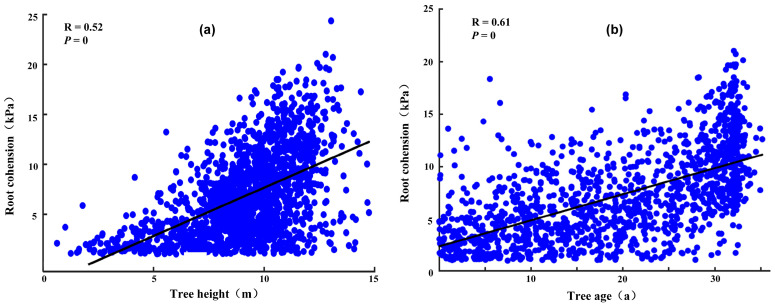
Relationship between root cohesion and tree height (**a**) and tree age (**b**).

Figure 5 shows the relationship between root cohesion and tree height and age. Figure 5a illustrates the relationship between root cohesion and tree height, revealing a moderate but highly significant positive correlation (R = 0.52, *p* < 0.001). Root cohesion increases with tree height, although the rate of increase diminishes beyond a threshold of approximately 15 m. In the lower height range (0–15 m), root cohesion rises rapidly, likely reflecting the priority of root development during early growth stages to support mechanical anchorage and resource uptake for above-ground biomass accumulation. However, beyond 15 m, the growth of root cohesion tends to plateau and data become more dispersed. This may be attributed to biological and ecological constraints: mature trees tend to allocate more resources to canopy expansion, reproduction, and maintenance respiration rather than further root reinforcement [50]. Additionally, variations in soil properties, water availability, nutrient conditions, and species-specific root architecture contribute to the increased variability in root cohesion among taller trees. This dispersion underscores the influence of both biotic and abiotic factors on root mechanical properties at later stages of tree development [51]. For instance, shallow-rooted species like cedar develop underdeveloped taproots with most roots near the soil surface, while deep-rooted species like fir possess taproots that can extend to depths of 3–5 m or even more than 10 m, offering greater slope stability. The stabilization of root cohesion at higher tree heights suggests an equilibrium or optimal point for root growth. Excessive root development beyond this point may divert resources that could hinder overall tree growth.

Root cohesion exhibits a moderately strong positive correlation with tree age (R = 0.61, *p* < 0.001), increasing significantly as trees mature, as shown in Figure 5b. In early developmental stages, rapid taproot growth predominates, while in intermediate stages, the expansion of lateral roots enhances root–soil contact. In mature trees, although overall root growth slows, lateral roots continue to extend, increasing frictional resistance and stabilizing the soil through a more intricate root network. Additionally, roots exude organic acids and enzymes that promote soil aggregation and stimulate microbial activity, further improving cohesion. While the root cohesion estimations in this study are derived from remote sensing–based allometric relationships, previous studies have demonstrated similar age-related root development trends across various broadleaf and coniferous species [52,53]. This general consistency supports the ecological plausibility of the proposed approach. However, some outliers in our data indicate that species-specific traits, soil types, and local climate conditions may also influence root development, underscoring the need for future field validation to refine species-level parameterization.

**Figure 6:**
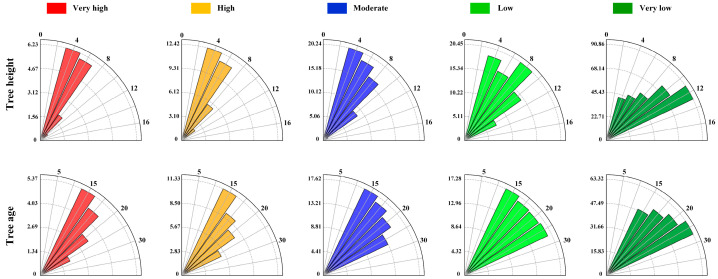
Distribution of tree height and age in different landslide susceptibility areas.

Figure 6 illustrates the distribution of tree height and age across different landslide susceptibility zones. It can be observed that in the study area, 51.46% of the trees are below 7.5 m in height. As tree height increases, the area classified as very low susceptibility also expands. Specifically, when tree height is less than 7.5 m, 47.72% of the area falls within the very low susceptibility zone, while 5.88% is classified as very high susceptibility. As tree height increases, the area of very low susceptibility continues to rise, reaching 90.86% when tree height exceeds 12.5 m. Conversely, the very high susceptibility zone decreases with increasing tree height, dropping to 0.18% when tree height surpasses 12.5 m. This suggests that, within a certain height range, greater tree heights are associated with reduced landslide susceptibility, assuming broadly similar lithological and soil distribution conditions. Furthermore, 63% of the trees in the study area are younger than 15 years, with only 3% falling between 15 and 25 years old. In the case of trees under 15 years old, 53.38% of the area is classified as very low susceptibility, while 4.62% is classified as very high susceptibility. As tree age increases, the very low susceptibility area expands, reaching 63.32% for trees older than 25 years. Conversely, the very high susceptibility zone decreases with tree age, accounting for only 1.84% when tree age exceeds 25 years. This trend indicates that older trees are less likely to contribute to landslides, assuming broadly similar lithological and surface soil conditions across the study area.

### 4.2. Validation of Root Cohesion Inversion Accounting for Spatial Heterogeneity

**Figure 7:**
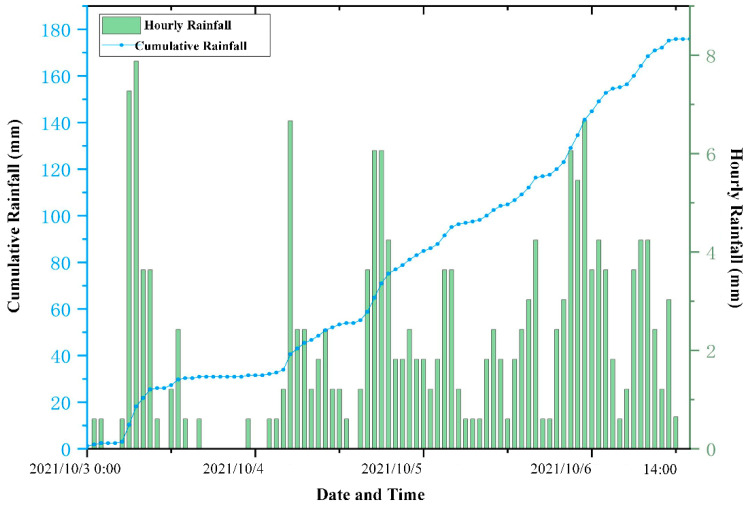
Hourly and cumulative rainfall in the study area from 00:00 on 3 October to 14:00 on 6 October 2021.

To validate the advantages of the root cohesion inversion model presented in this study, three scenarios were established: (1) Scenario I: Root cohesion is not considered. (2) Scenario II: Root cohesion values for different vegetation types are assigned based on previous research on root cohesion in the Loess Plateau, with values set as 0.15 kPa for arable land, 5 kPa for grassland, and 20 kPa for forest land. (3) Scenario III: Root cohesion is derived using the proposed inversion model, which estimates root cohesion based on below-ground biomass and tree height. All scenarios were evaluated using consistent soil and hydrological parameters, along with hourly rainfall data recorded from 00:00 on 3 October to 14:00 on 6 October 2021. The rainfall time series, shown in Figure 7, captures both hourly intensity and cumulative totals and was critical for simulating rainfall-induced landslide processes within the model.

**Figure 8:**
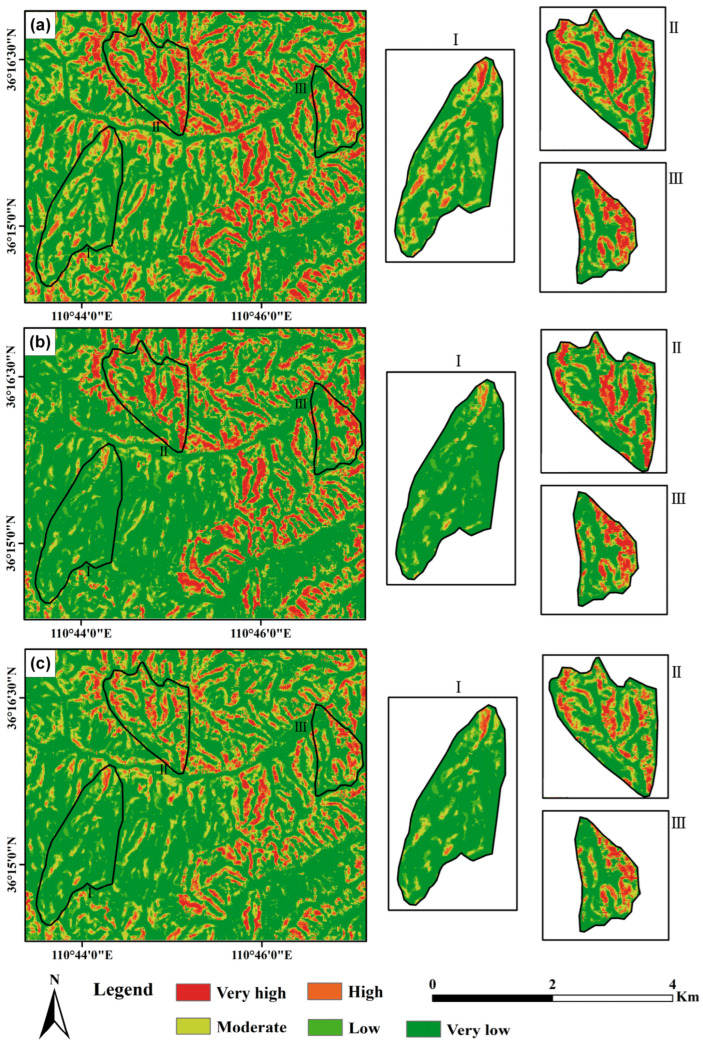
Landslide susceptibility maps for (**a**) Scenario 1, (**b**) Scenario 2, and (**c**) Scenario 3.

**Figure 9:**
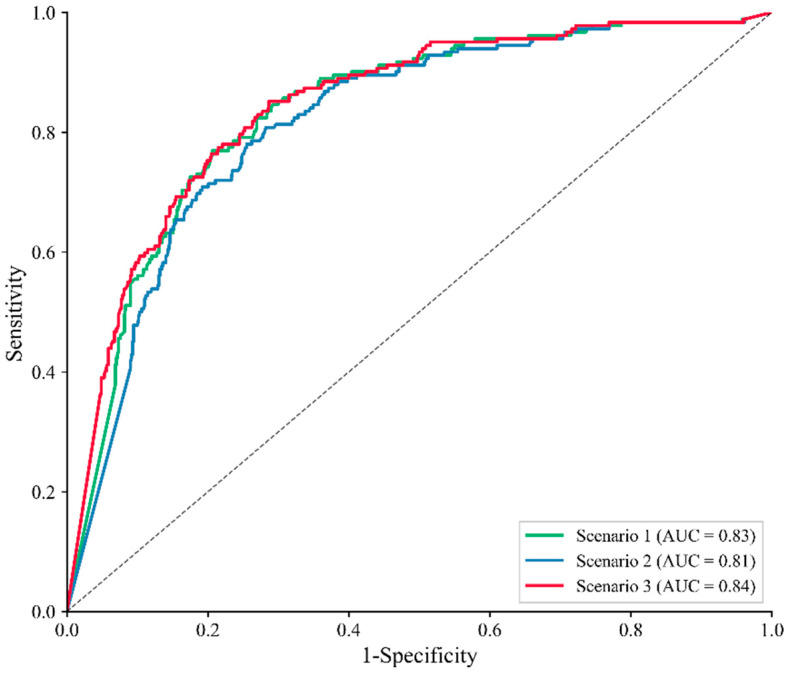
ROC curves for landslide susceptibility maps for (**a**) Scenario 1, (**b**) Scenario 2, and (**c**) Scenario 3.

Figure 8 and Figure 9 show the landslide susceptibility maps and corresponding ROC curves under the three defined scenarios. The susceptibility results were derived using an infinite slope stability model, with key input variables including soil cohesion, root cohesion, slope angle, soil internal friction angle, and rainfall. Susceptibility zoning was determined based on calculated stability values. In Scenario III, root cohesion was estimated using the proposed inversion model, whereas Scenarios I and II employed either no root cohesion or fixed values obtained from previous literature, respectively. As shown in Figure 8, high landslide susceptibility areas are predominantly distributed in regions with steeper slopes. The figure indicates that areas of extremely high susceptibility occupy a larger proportion of the agricultural land sub-watershed compared to the planted forest and secondary forest sub-watersheds. Additionally, the proportion of extremely high susceptibility areas is greater in the planted forest sub-watershed than in the secondary forest sub-watershed. Under varying land use conditions, landslide-prone areas in the agricultural land sub-watershed are primarily concentrated along gully slopes and agricultural land. In the planted forest sub-watershed, these areas form a band along the northwest-southeast ridgeline. By contrast, the secondary forest sub-watershed exhibits the smallest area of extremely high landslide susceptibility, primarily localized on slopes at valley intersections. Furthermore, compared to the other two root cohesion assignment methods, neglecting root cohesion results in a larger proportion of areas being classified as high landslide susceptibility zones, particularly in Sub-Area I.

To evaluate the accuracy of landslide susceptibility assessments under different vegetation root cohesion assumptions, both ROC (Receiver Operating Characteristic) curves and Frequency Ratio-based indicators were employed. As shown in Figure 9, Scenario 1 (without root cohesion) achieved an AUC of 0.83; Scenario 2 (with uniform root cohesion assignment) produced an AUC of 0.81; and Scenario 3 (with inverted root cohesion) reached the highest AUC of 0.84. These results indicate that detailed spatial modeling of root cohesion improves model performance.

In addition, the landslide ratio (LR) and normalized percentage landslide ratio (%LR) were used to further validate model performance across susceptibility zones. As shown in Table 4, scenario 3 consistently achieved the highest LR and %LR in the “very high” and “high” susceptibility classes (LR = 2.5897 and 1.6947; %LR = 36.66% and 23.99%), indicating a more accurate concentration of actual landslides in predicted high-risk areas. Compared to Scenarios 1 and 2, Scenario 3 showed a clearer distinction between risk levels and better matched the spatial distribution of observed landslides, further demonstrating the superiority of the inversion-based root cohesion model.

These results highlight that a detailed consideration of vegetation root cohesion not only enhances the accuracy of landslide susceptibility assessments but also leads to more reliable susceptibility zoning, emphasizing the value of precise modeling approaches.

### 4.3. Dynamics of Landslide Susceptibility Across Different Land Uses During a Rainfall Event

While previous sections focused on the evaluation of landslide susceptibility under static conditions—validating the effectiveness of root cohesion modeling and parameter configurations—real-world landslides often occur under dynamically changing environmental factors, particularly during rainfall events. To bridge the gap between static validation and actual landslide-triggering conditions, this section introduces a time-resolved susceptibility analysis that captures the evolving response of different land use types to rainfall.

**Figure 10:**
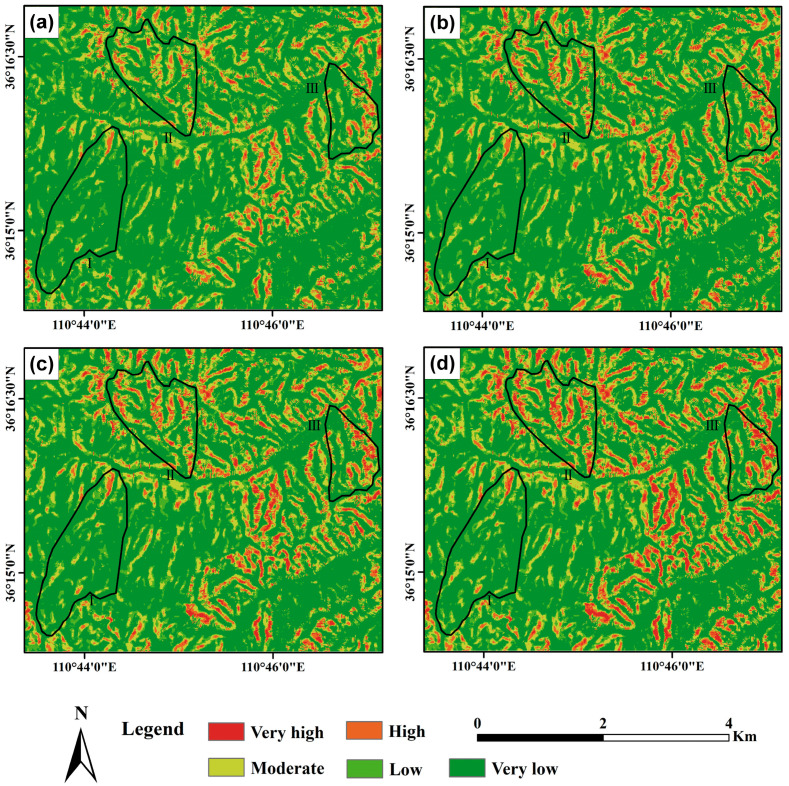
Landslide susceptibility maps for four representative time points: (**a**) 3 October 2021 0:00, (**b**) 4 October 2021 12:00, (**c**) 5 October 2021 12:00, and (**d**) 6 October 2021 12:00.

To investigate the dynamic characteristics of landslide susceptibility during a rainfall event, we selected four representative time points: (a) the initial onset of rainfall, represented by the susceptibility map at 00:00 on 3 October 2021; (b) the early stage of continuous heavy rainfall, at 12:00 on 4 October 2021; (c) the 24 h window preceding the end of rainfall, at 12:00 on 5 October 2021; and (d) the conclusion of the rainfall event, at 12:00 on 6 October 2021. These time points were chosen to reflect changes in rainfall intensity and duration and their effects on slope stability across different land use types. Figure 10 presents the landslide susceptibility maps for these four moments, revealing the spatiotemporal evolution of hazard levels throughout the event.

**Figure 11:**
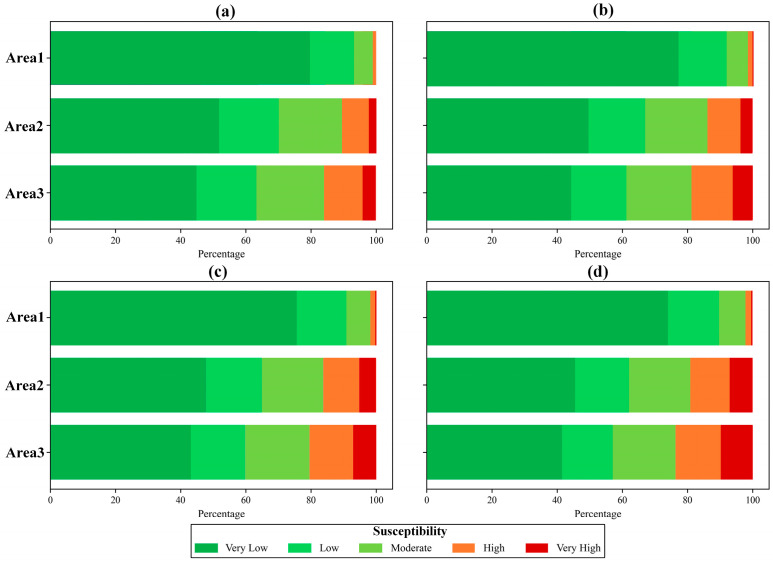
Percentage of landslide susceptibility in three sub-regions at four representative time points: (**a**) 3 October 2021 0:00, (**b**) 4 October 2021 12:00, (**c**) 5 October 2021 12:00, and (**d**) 6 October 2021 12:00.

Figure 11 illustrates the percentage of different landslide susceptibility levels in three sub-regions at four representative time points in Figure 10. It can be seen that landslide susceptibility in secondary forest (Area 3) varies across rainfall stages but remains relatively low due to dense vegetation and stable soil. In the initial stage (low rainfall, Figure 10 and Figure 11a), susceptibility remains minimal. As rainfall increases (Figure 10 and Figure 11b,c), susceptibility may rise slightly, though vegetation and soil cohesion prevent major escalation. Under extreme rainfall (Figure 10 and Figure 11d), soil saturation can gradually increase susceptibility, yet strong root systems help maintain stability, limiting high-risk zones. Plantation forest (Area 2) shows greater susceptibility than secondary forest. In the initial stage (Figure 10 and Figure 11a), weaker soil and sparser vegetation result in mixed low to moderate susceptibility. As rainfall intensifies (Figure 10 and Figure 11b,c), susceptibility increases due to monoculture plantations and weaker root systems, leading to more high-susceptibility zones. Under extreme rainfall (Figure 10 and Figure 11d), soil saturation sharply elevates susceptibility, with extensive high-risk areas appearing, highlighting the vulnerability of plantation forests. Agricultural land (Area 1) exhibits the highest susceptibility due to its unstable soil. Even under low rainfall (Figure 10 and Figure 11a), susceptibility is moderate to high, particularly in farmed or reclaimed areas. As rainfall intensifies (Figure 10 and Figure 11b,c), loosened soil and limited vegetation lead to a rapid increase in high-susceptibility zones. Under extreme rainfall (Figure 10 and Figure 11d), widespread high-risk areas emerge, especially on steep slopes, emphasizing the significant landslide hazard associated with agricultural land. Therefore, with the increase in precipitation, the susceptibility to landslides in secondary forests, plantations, and agricultural lands exhibits distinct variations, as secondary forests remain relatively stable with lower landslide susceptibility, showing minimal changes even with increased rainfall, while plantations may experience a gradual increase in landslide susceptibility, particularly during the intermediate stages of precipitation, and agricultural lands demonstrate a significant rise in landslide susceptibility, especially in areas with loose soil or steeper slopes. These variations are closely linked to factors such as soil stability, vegetation cover, and land use practices, which collectively influence landslide risk differently under varying rainfall intensities, highlighting the complex interplay between environmental conditions and human activities in shaping landslide dynamics.

### 4.4. Impact of Root Cohesion on Rainfall Thresholds for Landslides

**Figure 12:**
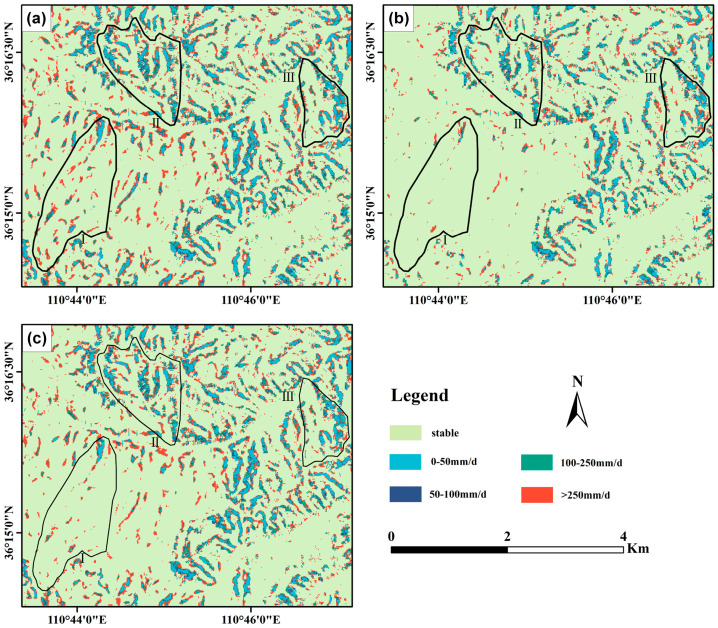
Spatial distribution of daily rainfall thresholds triggering slope instability under three root cohesion assumptions: (**a**) no root cohesion, (**b**) uniform assigned values, and (**c**) spatially varying inverted values.

Figure 12 shows landslide rainfall threshold maps for three root cohesion scenarios. The impact of root force assumptions on landslide susceptibility exhibits distinct patterns across three scenarios. (a) Zero root force assumption: When vegetation reinforcement is ignored, treating soil as structurally unsupported, landslide susceptibility is predominantly governed by terrain slope and rainfall intensity. Under this scenario, all three land use types exhibit extensive high-susceptibility zones. Agricultural lands, with loose soil and sparse vegetation, are most prone to failure, even under low rainfall conditions (0–50 mm/day). Plantations, while comparatively more stable, experience a sharp increase in susceptibility with rising rainfall. Secondary forests, though generally less vulnerable, still develop localized instability under intense precipitation. (b) Assigned root force assumption: This scenario applies a uniform root strength distribution, disregarding spatial and temporal variability, resulting in a “patch effect” in forested areas, where instability patterns appear artificially discontinuous. Agricultural lands remain highly susceptible, particularly on slopes under light rainfall (0–50 mm/day). Plantations exhibit reduced risk but retain scattered unstable zones due to the oversimplified root strength allocation. Secondary forests, while largely stable, also display discontinuous unstable patches, reflecting the limitations of uniform root force representation. (c) Inverted root force assumption: By capturing the spatial heterogeneity of root reinforcement, this approach yields susceptibility patterns more consistent with observed landslide distributions. Agricultural lands remain highly vulnerable, even under low rainfall. Plantations exhibit improved stability under moderate rainfall (50–100 mm/day), though susceptibility rises sharply beyond 100 mm/day. Secondary forests demonstrate the highest resilience, maintaining low susceptibility below 100 mm/day and only developing risk hotspots when rainfall exceeds 250 mm/day, underscoring the stabilizing role of mature vegetation. These findings highlight the critical influence of root force representation in landslide susceptibility assessments and its varying impacts across land use types under changing rainfall conditions.

The advantages of inverted root force modeling are evident in three key aspects. First, the inverted root reinforcement model effectively captures the spatial variability of root cohesion by integrating multiple factors: vegetation type (secondary forests, plantations, and agricultural lands), tree species, age, and root distribution depth. This approach avoids the patchiness inherent in assigned root cohesion models and reveals significant differences in root reinforcement across land use types. Second, the model enables a more realistic response to rainfall variability. Under low rainfall intensities (0–50 mm/day), it effectively differentiates the high stability of secondary forests from the increased susceptibility of agricultural lands. Under intense rainfall (>250 mm/day), it captures the progressive weakening of root reinforcement in plantations and secondary forests, avoiding the oversimplification of static stability. Third, the model accounts for coupled interactions among vegetation effects, terrain slope, and rainfall intensity, enhancing the reliability of landslide susceptibility predictions.

A comparative analysis of the three root reinforcement scenarios yields key insights. The zero root reinforcement assumption substantially overestimates landslide susceptibility by neglecting vegetation reinforcement. The assigned root reinforcement approach incorporates vegetation effects but fails to represent their spatial and temporal variability, resulting in unrealistic susceptibility patterns. In contrast, the inverted root reinforcement model, integrating remote sensing observations with process-based modeling, provides a more accurate and dynamic representation of forest root reinforcement and its response to rainfall variability.

Nevertheless, inherent uncertainties in this modeling approach must be acknowledged. Its heavy reliance on satellite-derived inputs means accuracy may be influenced by factors such as sensor resolution, classification accuracy, and atmospheric conditions. Furthermore, although species-specific vegetation parameters are incorporated, actual root development is highly variable in natural settings and influenced by multiple environmental conditions—including soil type, moisture, and competition—which cannot be fully captured in a regional-scale model. Thus, while the inverted root reinforcement model demonstrates clear improvements, its predictions represent probabilistic estimates rather than absolute outcomes, necessitating further calibration with field data.

## 5. Discussion

### 5.1. Uncertainties in Satellite-Derived Root Cohesion Models for Landslide Susceptibility Assessment

Our regional root cohesion inversion model, developed using Landsat, Sentinel-2 and GaoFen satellite imagery, enhances landslide susceptibility mapping across the Loess Plateau by capturing the spatial heterogeneity of root cohesion. Nonetheless, we acknowledge several sources of uncertainty inherent in this approach. Satellite-derived proxies such as tree height and above-ground biomass are influenced by limitations in spatial resolution, temporal variability, and environmental factors including soil properties, moisture availability, and micro-topographic variation. These factors affect root system development and contribute to uncertainties in estimating root cohesion. Even when tree species are well characterized, subsurface variability in root architecture [54] and soil–root interactions [55] further constrain the accuracy of our estimates, as satellite observations can only approximate the complexity of belowground systems. Additional uncertainties arise from other parameters in the infinite slope stability model [56], including internal friction angle, soil cohesion, and saturated hydraulic conductivity. These parameters are often derived from regional datasets or generalized assumptions, which may not accurately capture site-specific conditions and can compound overall model uncertainty.

Despite these limitations, our integration of high-resolution remote sensing data with land use-specific hydrological parameters represents a significant improvement over models that oversimplify or neglect root cohesion. The agreement between model predictions and validated landslide inventories demonstrates the practical utility of our approach for regional-scale risk assessment. Future research should focus on reducing uncertainties by combining ground-based measurements of root and soil properties with satellite-derived estimates, applying advanced deep learning techniques [57], and incorporating probabilistic frameworks to better account for parameter variability. These enhancements will improve the robustness of landslide susceptibility predictions and support more effective land use planning and disaster mitigation.

### 5.2. Complex Role of Root Cohesion in Slope Stability Under Various Conditions

Although this study highlights the importance of spatial heterogeneity in root cohesion for improving landslide susceptibility predictions on the Loess Plateau, the influence of root systems on slope stability is complex and context-dependent [58]. Roots generally enhance soil strength through cohesion; their stabilizing effect, however, can be substantially reduced under saturated conditions. Soil saturation decreases root tensile strength and weakens soil–root frictional interactions, thereby diminishing the reinforcement effect [59]. At the same time, vegetation, especially trees, contributes additional surcharge loads to slopes, which may increase the risk of slope failure during intense rainfall events. Furthermore, root systems can create preferential pathways for water infiltration [60], elevating pore water pressure and reducing shear strength, which may further destabilize slopes. While root cohesion plays a critical role in preventing shallow landslides by anchoring near-surface soil layers, its effectiveness in mitigating deep-seated landslides is limited, as roots typically do not extend to the depths of deeper failure planes [61].

These complexities underscore the need for more nuanced and dynamic modeling approaches. Our current model, which integrates satellite-derived root cohesion with land use-specific hydrological parameters, captures some of these interactions but does not yet account for the effects of soil saturation, vegetation surcharge, or root conductivity. Despite these limitations, our approach represents a significant improvement over models that oversimplify vegetation–slope interactions and offers valuable insights for shallow landslide risk assessment. Future work should incorporate dynamic soil moisture data and biomechanical modeling to better quantify the dual role of vegetation in slope stability, thereby improving predictions for both shallow and deep-seated landslides on the Loess Plateau.

## 6. Conclusions

This study proposes a novel framework integrating remote sensing-derived vegetation parameters into a physically based slope stability model for rainfall-induced landslide susceptibility assessment. The main conclusions are as follows:

(1) Root cohesion spatial heterogeneity is critical: Inversion of root cohesion using satellite-derived tree height and biomass revealed significant spatial variability. Secondary forests exhibited substantially higher cohesion values than farmland, attributable to greater tree height and stand age. This underscores the limitation of uniform root parameter assumptions.
(2) Spatially explicit cohesion mapping enhances validation: Using high-resolution Gaofen imagery to construct a validated landslide inventory, the model incorporating heterogeneous root cohesion achieved superior prediction accuracy. This significantly outperformed models assuming uniform root cohesion or neglecting root reinforcement, confirming the value of remote sensing-based inversion for susceptibility assessment.
(3) Land use governs dynamic susceptibility: Time-resolved analysis during an extreme rainfall event (>250 mm/day) identified farmland as the most vulnerable land use, with susceptibility escalating markedly during peak rainfall. Secondary forests exhibited greater resilience, attributed to their well-developed root systems.
(4) Integrated approach enables scalable assessment: Combining satellite data with physics-based modeling provides a scalable and transferable methodology for landslide susceptibility mapping, particularly valuable in data-scarce or inaccessible regions.

Despite these demonstrated advantages, several limitations remain. The spatial resolution of input vegetation data is limited to 30 m due to reliance on publicly available satellite imagery, potentially restricting the model’s ability to capture fine-scale variability in root cohesion, especially in heterogeneous or complex terrain. Furthermore, the model does not fully represent complex subsurface variability in root architecture and soil–root interactions, which are difficult to infer accurately from remote sensing proxies. Additionally, important hydromechanical processes—such as reduction of root reinforcement under saturated conditions, vegetation-induced surcharge loads, and preferential flow paths created by roots—are not explicitly incorporated, although they can influence slope stability.

Future research should aim to overcome these limitations by integrating higher-resolution vegetation metrics derived from UAV-LiDAR and multi-sensor data fusion, combining satellite estimates with ground-based measurements, incorporating dynamic soil moisture and hydrological data, and developing coupled biomechanical models that better represent vegetation’s dual role in slope stability. Such advances will enhance the precision, robustness, and applicability of landslide susceptibility assessments, especially in complex and data-scarce environments like the Loess Plateau.

Collectively, these findings highlight the necessity of incorporating vegetation structure and its spatial heterogeneity into landslide modeling and advocate for evidence-based land management strategies in landslide-prone environments.

## Figures and Tables

**Table 1:** Data sources used in this study.

Data Type	Format	Spatial Resolution	Source
DEM	Raster	12.5 m × 12.5 m	https://search.asf.alaska.edu/#/?zoom=3&center=125.336020,22.365939, accessed on 8 May 2024
Soil depth	Raster	1 km × 1 km	https://daac.ornl.gov/SOILS/guides/Global_Soil_Regolith_Sediment.html, accessed on 8 May 2024
Soil type	Raster	1 km × 1 k m	https://www.fao.org/soils-portal/data-hub/soil-maps-and-databases/harmonized-world-soil-database-v12/en/, accessed on 8 May 2024
Land use	Raster	30 m × 30 m	https://zenodo.org/records/5816591, accessed on 8 May 2024
Above ground biomass	Raster	30 m × 30 m	https://www.scidb.cn/en/detail?dataSetId=f48c4983dbd84a4c9c287111ac91c5aa, accessed on 8 May 2024
Tree height	Raster	30 m × 30 m	https://www.3decology.org/2023/06/21/forest-tree-height-map-of-china-2/, accessed on 8 May 2024
Tree age	Raster	30 m × 30 m	https://figshare.com/articles/dataset/CHINA_FAGE_30m_7z/21627023/7, accessed on 8 May 2024

**Table 2:** The saturated hydraulic conductivity for different land uses [48].

Land Use	Forest	Grassland	Bare Land
$K_{S}$ (m/d)	2.4192	0.792	0.144

**Table 3:** Geotechnical parameters for each soil type [49].

Soil Type	$C_{s}$ (kPa)		$\phi^{\prime }$ (°)	
	Min	Max	Min	Max
Loamy sand	10	20	31	34
Sandy loam	10	20	31	34
Loam	10	20	28	32
Silty (sandy) loam	10	20	25	32
Sandy clay loam	10	20	25	32
Sandy clay loam	10	20	31	34
Clay loam	10	20	18	32
Clay	10	20	18	28
Sandy clay	10	20	31	34
Silty (sandy) clay	10	20	18	32

**Table 4:** LR and %LR in different scenarios.

Scenario	Susceptibility	% of Predicted Areas	% of Landslide	LR	%LR
without root cohesion	Very high	7.1	16.61	2.3409	36.20994
	High	15.6	23.22	1.4885	23.02469
	Moderate	17.68	19.71	1.1149	17.2457
	Low	14.05	12.39	0.8821	13.64466
	Very low	43.74	27.92	0.6384	9.875015
with uniform root cohesion assignment	Very high	8.92	19.41	2.1772	33.0801
	High	10.77	17.11	1.5892	24.1461
	Moderate	14.02	17.25	1.2302	18.6915
	Low	13.87	12.92	0.9311	14.14702
	Very low	50.57	33.07	0.6539	9.935274
with inverted root cohesion	Very high	5.02	13.01	**2.5897**	**36.66001**
	High	13.28	22.51	**1.6947**	**23.99032**
	Moderate	16.8	20.14	1.1988	16.97031
	Low	14.69	14.07	0.958	13.56153
	Very low	48.36	30.12	0.6229	8.817825

## Data Availability

Data will be made available on request.
